# Symptom burden among patients with Renal Cell Carcinoma (RCC): content for a symptom index

**DOI:** 10.1186/1477-7525-5-34

**Published:** 2007-06-14

**Authors:** Gale Harding, David Cella, Don Robinson, Parthiv J Mahadevia, Jason Clark, Dennis A Revicki

**Affiliations:** 1 UBC Center for Health Outcomes Research, 7101 Wisconsin Ave., Suite 600, Bethesda MD, USA; 2 Evanston Northwestern Healthcare & Northwestern University Place, Suite 100, Evanston, IL, USA; 3 Outcomes Research, Centocor Research and Development, Inc., 200 Great Valley Parkway, Malvern, PA, USA

## Abstract

**Background:**

Renal cell carcinoma (RCC) has multiple symptoms stemming from disease and treatments. There are few validated scales for evaluating RCC symptoms.

**Methods:**

A national cross-sectional study of adult RCC patients was conducted from October to December 2003 to define patient-reported RCC symptomology. Participants were asked open-ended questions regarding their signs and symptoms and completed an 86-item pilot questionnaire of physical and psychological symptoms. Patients were asked to rate the relevancy and clarity of each pilot question using a 5-point Likert scale. Subsequent open-ended caregiver interviews and a provider panel relevance ranking contributed additional information.

**Results:**

The average age of the participants (n = 31) was 55 years; 55% of patients were male, 74% had attended college, and 97% were Caucasian. The five most frequent symptoms among localized-stage patients (n = 14) were irritability (79%), pain (71%), fatigue (71%), worry (71%), and sleep disturbance (64%). Among metastatic patients (n = 17), the five most frequent symptoms were fatigue (82%), weakness (65%), worry (65%), shortness of breath (53%), and irritability (53%). More than 50% of localized and metastatic-stage patients reported pain, weakness, fatigue, sleep disturbance, urinary frequency, worry, and mood disorders as being moderately to highly relevant.

**Conclusion:**

A brief, self-administered RCC Symptom Index was created that captures the relevant signs and symptoms of both localized and metastatic patients. Pending additional content validation, the Index can be used to assess the signs and symptoms of RCC and the clinical benefit resulting from RCC treatment.

## Background

Renal cell carcinoma (RCC) is the most common type of kidney cancer and comprises about 85% of all kidney tumors [1,2]. Approximately 30,600 patients are newly diagnosed each year with RCC in the United States (US), and an estimated 12,000 deaths occur annually from this malignancy [3]. At the time of diagnosis, patients are often in advanced stages of the disease, with 40% to 50% of patients presenting with unresectable or metastatic disease [4,5]. Treatment with biological agents, such as interleukin-2 alone or in combination with interferon-alpha, has demonstrated the highest survival benefit. These therapeutic approaches remain limited due to minimal prolongation of life and severe toxicities associated with treatment [6,7]. Despite recent advances in treatment options and the emergence of several experimental therapies, the prognosis for long-term survival remains low, with or without therapy. The 5-year cumulative survival rate of these patients ranges from 5% to 10% [5].

Given the toxic side effects associated with current treatment and the poor survival prognosis among patients with RCC, health-related quality of life (HRQL) has become an important medical outcome among this patient population. A number of clinical studies among patients with RCC have assessed symptoms and HRQL [8-19]. Along with survival, the FDA oncology division considers symptom improvement to be one of the primary measures of clinical benefit [20]. Consistent with other tumors [21], there is some evidence linking survival and symptoms in RCC that suggests an association between tumor and symptom burden [22-25]. A number of general cancer questionnaires have been used to evaluate RCC symptoms or HRQL from the patient perspective, including the Functional Assessment of Cancer Therapy-Biologic Response Modifier (FACT-BRM) [26], the Rotterdam Symptom Checklist [27], and the European Organization for Research and Treatment of Cancer HRQL survey (EORTC QLQ-C30) [28]. While these questionnaires were designed to assess HRQL among cancer patients, these measures are not specific to RCC or kidney cancer overall.

At the time of this study there was no published questionnaire developed to capture the symptoms specific to RCC or kidney cancer patients. While a number of general cancer symptom scales exist, their use among RCC patients may add to the respondent burden of patient-reported outcomes by asking about symptoms and problems that may not be relevant to this population. In addition, existing cancer questionnaires may not adequately capture all the patient symptoms associated with the disease. The purpose of this study was to identify the appropriate content for a disease-specific questionnaire for the assessment of symptom burden in RCC patients.

Our conceptual model for a RCC Symptom Index (Index) incorporated signs and symptoms, as classically defined [29,30]. Questions about the impact of these signs and symptoms on the activities of daily living were also included when clearly linked by the patient or physician to the disease. In this way, symptom-driven functional impairment was an indirect measure of symptom burden [31]. While the conceptual framework of a HRQL questionnaire is important, so too are its performance characteristics if it is to inform treating physicians and health policy makers about the clinical benefit of treatment. In this regard, we were guided by scientific conventions for HRQL questionnaire development [32,33].

To facilitate the standardization of HRQL assessment in cancer and promote HRQL comparison across treatment modalities, the authors used an established framework for questionnaire development: the Functional Assessment of Chronic Illness Therapy (FACIT) model [34]. The FACIT model focuses on the frequency of disease manifestations over a 7-day recall period. A short recall period is needed to ensure accurate patient recall of the symptom experience while avoiding the bias that could result from memories based on any one moment, since symptoms wax and wane. The FACIT model measures symptom frequency, rather than intensity or duration, which are two other key aspects of symptom assessment. Two of the most common kidney cancer symptoms, fatigue and pain, have demonstrated a close association between symptom frequency and intensity, as well as functional impact when using short patient recall periods [22,30,35-41]. Consequently, the 7-day recall of RCC symptom frequency is a meaningful measure of intensity, as well as functional impact, and was considered appropriate for the Index.

## Methods

### Study design

**Figure 1:**
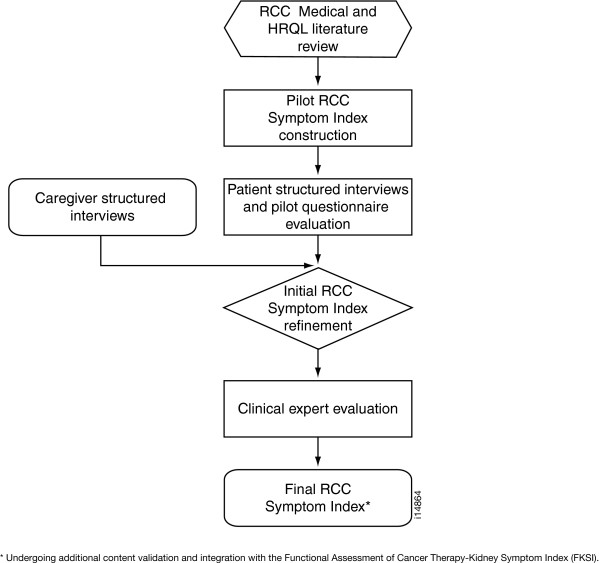
RCC Symptom Index Development Process.

A triangulation or convergence approach was used for content development of the Index [42]. Multiple sources of information, including qualitative and quantitative data, were pooled in an inclusive process that held patient opinion as primary for the identification of Index questions (Figure 1). Caregivers or providers could add items to the questionnaire, but not remove any items identified by patients as clear and relevant. Any question that was a source of investigator disagreement was added to the Index, with the understanding that subsequent construct validation research would clarify the performance of various items.

The first step in this process was a narrative review of the quality of life literature for RCC or kidney cancer patients. This literature review was the basis for constructing a pilot Index comprising relevant items from established cancer quality of life questionnaires. The second step was a national, cross-sectional, semi-structured telephone interview of adult RCC patients and their caregivers conducted from October to December 2003. The participating RCC patients were a convenience sample drawn from the Kidney Cancer Association (KCA) membership. The patient interviews began with a series of open-ended questions about their RCC signs and symptoms and ended with the patient completion of a self-administered pilot questionnaire. The qualitative component of the caregiver interviews were similarly conducted, but the evaluation of the pilot questionnaire items was not included in their telephone interviews.

The pilot questionnaire was revised using patient and caregiver interview findings and then evaluated by physicians and nurses who had experience with the RCC population (Figure 1). Descriptive statistics were used to characterize the socio-demographic and clinical characteristics of the RCC patient sample. Questions for the Index were selected based on the 1) frequency distributions from the patient relevance rankings, 2) patient comments on item clarity, 3) responses from open-ended patient and caregiver questions, 4) provider relevance rankings, and 5) investigator assessment of item clarity, redundancy and likely disease, rather than treatment, origin. Additional details on each step are provided in the following sections. Institutional Review Board approval was obtained to comply with human participant research requirements prior to study initiation. All participants gave written, informed consent before beginning study procedures.

### Literature review and pilot questionnaire creation

The PubMed literature database was used to identify potentially important signs, symptoms, and related HRQL issues among localized and metastatic RCC patients. The search selected RCC clinical studies that included HRQL, including those with only baseline symptom assessment. Studies that were not available in English, non-human in test subject, or included fewer than 20 patients were excluded from the literature review.

A pilot questionnaire was constructed using items from existing cancer HRQL questionnaires. Questions were selected for the pilot Index if the items specifically addressed the RCC signs and symptoms, along with associated functioning issues, that were identified in the literature review. An item's relevance to a RCC patient was based on their response to a standard Likert 5-level scale: not at all (0), a little bit (1), somewhat (2), quite a bit (3), very much (4).

### Patient interviews

The patient sample was recruited through the KCA, a nonprofit membership organization based in Chicago, IL. The KCA comprises patients, family members, physicians, researchers, and other health professionals involved in kidney cancer care. The KCA provides information on the latest research in kidney cancer and acts as a patient advocate with the federal government, insurance companies, and employers. Postcards were sent to 1,500 KCA mailing list members to recruit study participants. Potential study participants who responded by telephone to the postcard invitation were screened via telephone using a standardized form to ensure that participants met all entry criteria. To be eligible, participants 1) had a diagnosis of RCC or kidney cancer; 2) were 21 years of age or older; 3) were either previously treated or currently being treated for this condition; and 4) were able to speak and understand English. Study enrollment was stratified on self-reported localized or metastatic disease since Health Insurance Portability and Accountability Act of 1996 (HIPAA) regulations made access to patient medical charts difficult, further compounded by the geographically-dispersed nature of this national patient cohort. A variety of standard demographic and clinical questions were included in the interview to characterize the study population.

A patient sample of 30 was considered adequate for this content validation research based on qualitative research practices, as well as investigator experience [42]. RCC patients were mailed the pilot questionnaire in advance, to be completed during a scheduled telephone interview and later returned by mail. Professional research staff trained in qualitative interviewing techniques conducted the interviews. Patients were first asked open-ended questions about RCC symptoms they had experienced and how their daily life had been affected by these symptoms. This was done prior to completing the pilot questionnaire to maximize the discovery of new signs and symptoms that had not previously appeared in the literature. In addition, this minimized any content biasing the pilot questionnaire may introduce. In the open-ended portion of the interview patients were asked about their symptom experience at the time of initial diagnosis, prior to diagnosis, and after treatment. Patients were then asked to complete the pilot questionnaire. Patients who had undergone a nephrectomy were asked to use a recall period "just prior to surgery" to capture relevant symptoms since patients might become asymptomatic after their surgery [43-45].

After completing the pilot questionnaire, patients were asked about their overall assessment of the pilot questionnaire. The structured dialogue was designed to elicit the items most relevant to RCC patients since the pilot questionnaire was based on a synthesis of validated cancer HRQL scales. Besides rating the relevance of pilot questionnaire items, patients were asked to identify items that were unclear or confusing using the same 5-level Likert scale applied to relevance ranking. For multiple items that addressed the same or similar concept, patients were asked to identify the most clear and understandable item (i.e., the item that best represents or describes how they feel). Finally, patients were asked if there were any other signs, symptoms or related health issues they thought were important that were not included in the pilot questionnaire.

### Caregiver interviews

A convenience sample of caregivers was recruited from the participating RCC patients to gain additional information on the symptom burden they had observed. RCC patients were asked at the conclusion of their semi-structured interviews whether they had a family member or friend who would be willing to participate in a telephone interview. Potential caregiver study participants were screened over the telephone and had to meet the following eligibility criteria: 1) 21 years of age or older; 2) a family member or friend who sees or speaks to the RCC study participant at least 4 times a week or resides with him or her; and 3) provides care to RCC study participant on a self-reported, consistent basis. As with the patients, caregiver interviews focused on the symptoms that the person they cared for had experienced before, during, and after treatment. Caregivers were asked about the impact of these symptoms on the RCC patient's daily life, to describe the physical and emotional symptoms that bothered the RCC patient the most before and after treatment, and the changes that they noticed in signs, symptoms, and functioning once treatment began.

### Index item generation

A content analysis approach was used to evaluate the qualitative data collected from interviews. Three researchers independently reviewed the interview transcripts of RCC patients and caretakers to identify and summarize key themes, recurrent words, and specific health issues. This summary was used to identify the signs and symptoms relevant to patients with RCC.

Descriptive statistics (frequency of endorsement of each response option) were used to examine the patient relevance rankings of the pilot questionnaire by disease status: localized or metastatic. Sensitivity analysis explored different cut-points in item relevance for all patients as well as clinical subsets. Item exclusion was based on several criteria: 1) patient ratings of item relevancy; 2) understandability of items; and 3) item redundancy. Items identified by 50% or more of respondents as irrelevant (rating of 0 or 1) were deleted, as were items reported by 10% or more of patients as "unclear." Those questions rated by patients as "somewhat" to "very much" relevant were selected from the pilot questionnaire for inclusion in the Index.

### Provider assessment

The resulting initial Index was subsequently reviewed for comprehensiveness and clinical relevance by a panel of healthcare providers who were working in kidney cancer care. These experts were identified based on their familiarity with HRQL issues in kidney cancer and RCC patients. Each member of the panel reviewed the Index and rated the relevance of each item on a scale ranging from 0 (not at all relevant, delete) to 4 (highly relevant, must be included). In addition, clinicians were asked to note if any item was unclear and to provide suggested changes. Open-ended questions were also included to capture other important signs or symptoms that did not appear in the draft Index. Provider ratings of symptom relevance influenced the item list through the inclusion of questions, but never overruled patient valuation.

### Final item review

Study investigators assessed the Index item list after the provider panel evaluation to ensure that all the appropriate domains and items were adequately represented in the final Index. Additional items from the other sources: literature review, provider panel and caregiver observations were also considered for inclusion at this point. Questions that met the Index inclusion criteria for either the local or metastatic sub-populations of RCC patients were included without any weighing of Index domains.

## Results

### Literature review and pilot questionnaire generation

Twelve studies were selected for review, including two randomized controlled studies, two non-randomized comparative trials, and eight observational studies (Table 1). Based on findings from these studies, the most frequently reported symptoms among RCC patients include fatigue, weakness, pain, lack of appetite, nausea, dyspnea, flu-like symptoms, diarrhea, constipation, headache, and dry mouth. Results also suggest that patient HRQL is affected, particularly with respect to physical functioning, psychological impairment (depression, anxiety, irritability), sleep, social functioning, and role activities.

**Table 1:** Literature Review Summary Results for RCC Symptoms or HRQL

**Citation**	**Number**	**Study Design**	**Instrument**	**Results of Symptom and/or HRQL assessment**
MRC Renal Cancer 1999 [8]	N = 335	Randomized	Rotterdam Symptom Checklist	Most frequent symptoms at 4 weeks: tired (57%), lack of energy (53%), lack of appetite (33%), dry mouth (27%), nausea (15%), shivering (13%), heartburn (12%)
Motzer et al. 2000 [9]	N = 284	Randomized	FACT-BRM	QOL decreased from baseline to 8 weeks for treatment arms. Item analysis of FACT-BRM indicates items that address the following likely to be important at baseline: energy, pain, sex life, overall well-being, sleep, fatigue, appetite, energy, weakness (Cella D, personal communication, September 2003)
Capuron et al. 2001 [10]	N = 33	Non-randomized	MADRS	Positive correlation between depressive symptoms and the variation in the cytokine levels in the first week of therapy.
Heinzer et al. 1999 [11]	N = 20	Non-randomized	EORTC-QLQ-30	Most frequent symptoms: fatigue (29%), nausea (23%), cough (16%).
Atzpodien et al. 2003 [12]	N = 22	Observational	SF-36 EORTC-QLQ-30	Significant worsening in physical, social, and role functioning. Significant worsening in symptoms of appetite loss, nausea/vomiting, sleep disturbance, diarrhea, pain
Bukowski et al. 2002 [13]	N = 70	Observational	AE events	Most frequently reported adverse events over 1 year: fatigue (38%), anorexia (34%), pain (34%), headache (31%), myalgia (28%), weight loss (28%), nausea (24%), alopecia (21%), coughing: (21%), dyspnea (21%), fever (21%), rigors (21%)
Cohen et al. 2002 [14]	N = 36	Observational	SF-36	Changes from baseline to 3 weeks indicate improvement in physical functioning, role limitations (physical), bodily pain, vitality, and social functioning
Joffe et al. 1996 [15]	N = 55	Observational	Rotterdam Symptom Checklist	During 8-week treatment cycle, significant worsening in symptoms: loss of appetite, dry mouth, lack of energy, feeling nervous, lack of sexual interest, shivering, nausea, tiredness
Naglieri et al. 2002 [16]	N = 42	Observational	Graded toxicities	Total prevalence: fatigue (100%), cutaneous erythema (100%), fever (76%), anemia (10%), hypotension (5%), nausea/vomitting (2%), fluid retention (2%)
Shamash et al. 2003 [17]	N = 33	Observational	EORTC	Mean baseline scores indicate greatest impairment in pain, sleep, weakness, daily activities, tired, leisure/social
Stark et al. 2002 [18]	N = 178	Observational	HAD-A EORTC-QLQ-30	Patients diagnosed with anxiety had higher scores for symptoms of fatigue, nausea, insomnia, appetite loss, constipation, diarrhea
Whitehead et al. 2002 [19]	N = 37	Observational	Graded toxicity	Most common side effects (% with any grade toxicity): nausea/vomiting/diarrhea: 88%, headache/pain: 82%, malaise/fatigue/lethargy: 78%, fever/chills: 57%, edema: 51%, CNS: 43%, anemia/bleeding: 33%, myalgia/arthralgia: 31%, renal: 29%, pulmonary: 27%, rash/urticaria: 24%, hypertension: 8%, cardiac: 4%, hypotension: 4%

**Figure 2:**
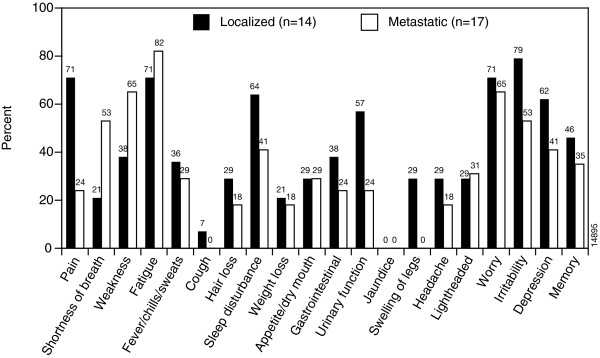
**Pilot RCC Questionnaire Domain Relevance Frequency**. Domain relevance was based on patients rating at least one item within a sign or symptom domain as "Somewhat" to "Very much" relevant to their lives. Percentages listed above each domain use all patients as the denominator (localized and metastatic). Not all domains were included into the final Index due to the importance rankings of each question within the domain.

The pilot questionnaire resulting from this process was 86 items in length and covered 20 domains or health issues of interest (Figure 2). Questions were included from the Functional Assessment of Cancer Therapy-General (FACT-General) [46] as well as items from the prostate cancer (FACT-P) [47], lung cancer (FACT-L) [48], fatigue (FACT-F) [49,50], hepatobiliary cancer (FACT-HEP) [51], and biological response modifier (FACT-BRM) [26] FACT subscales. Items were also included from the European Organization for Research and Treatment of Cancer HRQL survey, the EORTC-QLQ-C30 [28].

### Patient and caregiver cohort selection

Patient entry into the study sample was stopped at 162 patients, although inbound telephone calls expressing interest in the study continued. The first 46 respondents who were successfully contacted by telephone were screened for study entry. All but three met eligibility criteria since the criteria were broad and the recruitment process closely matched the entry criteria. The first 31 patients from the pool of 43 who were approached entered into the study without exception.

Among the RCC patients, 55% (n = 17) were male and 97% (n = 30) were Caucasian. Approximately one-third (32%, n = 10) of the RCC study sample reported that they were employed full-time, while the same number (32%, n = 10) reported that they were disabled, and 26% (n = 8) were retired. Almost all of the RCC patients had a high school education or higher, with nearly half (48%) reporting a college or postgraduate degree. The year of diagnosis ranged from 9 years prior to within 1 year of study initiation (Table 2).

**Table 2:** Demographics of RCC Patients

**Characteristic**	**N = 31**
**Age, mean (SD)**	54.6 (11.4)
**Gender, n (%)**	
Male	17 (54.8)
Female	14 (45.2)
**Race, n (%)**	
White	30 (96.8)
Black or African American	1 (3.2)
**Employment Status, n (%)**	
Full-time	10 (32.3)
Part-time	1 (3.2)
Retired	8 (25.8)
Disabled	10 (32.3)
Other	2 (6.5)
**Highest Education Level, n (%)**	
Elementary	0 (0)
High School	6 (19.4)
Some College	8 (25.8)
College degree	9 (29.0)
Postgraduate degree	6 (19.4)
Other	2 (6.5)
**RCC spread, n (%)**	
Localized	14 (45.2)
Metastatic	17 (54.8)
**Range of diagnosis year**	1994–2003

All data were patient-reported at time of interview. Percentages may not equal 100 due to rounding.

Among study patients, 45% (n = 14) reported localized-staged disease and 55% (n = 17) reported that their cancer had metastasized. The mean length of time since last treatment was 9 months and 6 months for localized and metastatic-staged patients, respectively (Table 3). Of those with self-reported metastatic disease, 47% (8/17) indicated that their last treatment included biologic agents. The caregivers (n = 10) in the study had a mean age of 52, all were Caucasian and half were female. All but one caregiver had more than a high school education; 5 were employed full time (data not shown).

**Table 3:** Clinical Characteristic of RCC Patients, by Localized and Metastatic-Staged Disease

	**Localized (n = 14)**	**Metastatic (n = 17)**
**Year of diagnosis, n (%)**		
2003	6 (42.9)	3 (17.6)
2002	8 (57.1)	5 (29.4)
2001	0 (0.0)	4 (23.5)
2000	0 (0.0)	2 (11.8)
1999 or earlier	0 (0.0)	3 (17.6)
**Last treatment, n (%)**		
Surgery	14 (100)	6 (35.3)
Biological agents	0 (0.0)	8 (47.1)
Chemotherapy	0 (0.0)	1 (5.9)
Other	0 (0.0)	2 (11.8)
**Months since last treatment: mean (SD),**	8.6 (4.5)	6.0 (14.1)
**median, [range]**	8.5 [3.0–15.0]	1.0 [0.0–58.0]

All data patient-reported at time of interview. Percentages may not equal 100 due to rounding.

### Patient and caregiver interviews

All 31 patients and 10 caregivers were interviewed. Based on their qualitative responses, the three most common reasons for seeking medical attention included pain in abdomen or back area, fatigue, and urinary problems including hematuria and passing of clots. The onset of pain varied from sudden onset to ongoing feelings of pain, with varying levels of pain severity described. Fatigue was described as "having very little energy," "exhausted," and "real severe fatigue." Other symptoms mentioned less often and classified as being present prior to diagnosis, included diarrhea, constipation, weight loss, and nausea. In general, patients with metastatic RCC focused more on being tired and experiencing noticeable weight loss than patients with localized-stage RCC when describing the symptoms that led them to seek medical attention.

While RCC patients felt that their daily and leisure activities were limited by symptoms, most addressed the emotional experience more than the physical, with emphasis on depression and worry. This emotional impairment was due in large part to initial misdiagnoses and continuing symptoms. Post-treatment, nearly all patients with localized disease mentioned that they still experienced pain subsequent to a nephrectomy, although the pain severity had decreased after surgery. Those with metastatic disease reported flu-like symptoms associated with treatment. Caregiver interviews closely mirrored patient interviews with respect to physical symptoms as they related to RCC, with pain being the predominant symptom. As expected, there was more divergence between RCC patient and caregiver views with respect to the patients' emotional symptoms such as depression, although no clear patterns were apparent.

### RCC symptom index

All 31 RCC patients completed and returned the pilot questionnaire. The symptom profile of these RCC patients at the domain level using pilot questionnaire data appears in Figure 2. The five most frequently reported symptoms for which patients responded either "somewhat," "quite a bit," or "very much" among localized-staged patients included items on irritability (79%), pain (71%), fatigue (71%), worry (71%), and sleep disturbance (64%). Among metastatic patients, the five most frequently reported symptoms came from items reporting fatigue (82%), weakness (65%), worry (65%), shortness of breath (53%), and irritability (53%). Fewer than 25% of people with metastatic disease reported "somewhat, "quite a bit," or "very much" for any single pain item, possibly due to ongoing pain treatment. More than 50% of localized and metastatic-staged patients reported pain, weakness, fatigue, sleep disturbance, urinary frequency, worry, irritability, and depression as being moderately (a score of 2 on a scale of 0–4) to highly relevant (score of 3 or 4 on a scale of 0–4).

The Index item list was reduced from the initial 86 items based on patient and caregiver interviews along with patient relevance rankings. Only one item was deemed "not relevant" based on a mean score of "1" or less on a scale of 0–4 for both localized- and metastatic-staged patients. An additional 12 items were reported by 10% or more of study patients as difficult to understand and were therefore deleted. Items that met inclusion criteria were retained (Table 4). The provider panel of five physicians and five nurses confirmed all items that remained were moderately-to-highly relevant to this patient population, but also contributed several items that patients had not ranked as meeting the relevance threshold (Table 4).

**Table 4:** RCC Index Mean Relevancy Item Scores of RCC Patients and Clinicians^1^

**Item**	**Patient Ratings**			**Clinician Ratings**		
	
	**Localized**	**Metastatic**	**Total**	**Nurse**	**Physician**	**Total**
**PAIN**						
I have pain	2.4	1.8	2.1	3.8	4.0	3.9
Pain interfered with my daily activities	2.0	1.8	1.9	3.8	3.2	3.5
I have pain in my back	2.0	1.1	1.5	2.4	2.6	2.5
I have discomfort or pain in my stomach area	1.7	1.8	1.8	2.6	1.8	2.2
**BREATHING**						
I have been short of breath	0.7	2.5	1.7	3.8	3.0	3.3
**WEAKNESS**						
I have felt weak	2.2	2.8	2.5	4.0	3.6	3.8
**MOOD**						
I worry that my condition will get worse	3.0	3.1	3.1	3.6	2.4	3.0
I have emotional ups and downs	2.6	2.2	2.4	3.6	1.6	2.6
I feel depressed	2.6	2.0	2.3	3.6	1.8	2.7
I am able to enjoy life^2^	2.2	2.8	2.5	3.8	3.0	3.4
**SLEEP**						
I have had trouble sleeping	2.5	2.1	2.3	3.6	2.4	3.0
**WEIGHT LOSS**						
I am losing weight	1.4	2.0	1.7	3.5	3.6	3.6
**COGNITIVE**						
I have difficulty remembering things	1.6	1.6	1.6	3.5	2.0	2.7
I have trouble concentrating	1.7	2.1	1.9	3.5	2.0	2.7
**FATIGUE**						
I feel fatigued	2.3	2.0	2.2	3.8	3.8	3.8
I have a lack of energy	2.4	2.5	2.5	3.8	3.2	3.4
I feel tired	2.3	2.1	2.2	3.8	3.0	3.3
I have trouble starting things because I am tired	1.7	2.1	1.9	3.0	2.0	2.5
I have trouble finishing things because I am tired	1.9	2.4	2.2	3.0	2.0	2.5
**APPETITE**						
I have lacked appetite	1.5	1.9	1.7	3.6	3.6	3.6
I have a good appetite^3^	2.5	3.1	2.8	3.0	3.4	3.2
**INCONTINENCE (BOWEL OR BLADDER)**						
I have control of my bowels^4^	2.4	2.5	2.5	2.2	1.6	1.9
I have trouble moving my bowels	1.6	1.3	1.5	3.6	2.2	2.9
I urinate more frequently than usual^5^	1.7	1.7	1.7	2.2	1.6	1.9
I have difficulty urinating	1.6	1.2	1.4	2.4	1.6	2.0
**OTHER SYMPTOMS**						
I have had chills	1.4	1.8	1.6	3.4	2.6	3.0
I have had fevers	1.3	1.5	1.4	3.8	3.0	3.4
I have had sweats^6^	1.2	1.1	1.1	3.8	3.2	3.5
I feel lightheaded	1.6	1.7	1.6	2.8	2.6	2.7
I am bothered by blood in my urine^7^	n/a	n/a	n/a	2.0	2.8	2.4

^1 ^Relevancy scores based on a scale of 0–4, where 0 indicated "not at all " relevant and 4 indicated "very much" (or highly) relevant.

^2 ^"I am able to enjoy life" was slightly modified for clinicians for testing purposes; item tested was "I am content with the quality of my life right now".

^3 ^Not included due to redundancy with the other appetite item and incompatible direction of question phrasing (positive).

^4,5 ^Not included due to uncertainty regarding causation: disease or treatment, as well as biological rationale.

^6 ^Modified from "I am bothered by sweating" for clarity and consistency with other items.

^7 ^Relevancy ratings not available since item was included based on findings from patient interviews

Three questions were removed by the investigators, from the Index items listed in Table 4, during final item review. An appetite item was dropped since it was redundant and the direction of the phrasing was incompatible with the other questions in the Index, all of which inquired about declining health status. Another item on frequent urination was not included since it was linked in patient interviews to the result of surgery rather than the disease and the positive nurse scoring of this item could also reflect this treatment relationship. Similarly, an item on diarrhea was also removed since its biological rationale was suspect and poor bowel control could result from treatment. Diarrhea did appear in the RCC literature review, however, and in some patient interviews was described as occurring before diagnosis, although this involved a long recall period. Given the confounding factors in this study, further exploration of this issue was left for a follow-on content confirmation study. The resulting Index covers pain (4 items), breathing (1 item), weakness (1 item), mood (4 items), sleep (1 item), weight loss (1 item), cognitive functioning (2 items), fatigue (5 items), appetite (2 items), incontinence (2 items bowel and 2 items bladder), and other constitutional symptoms (5 items; Table 5).

**Table 5:** Final RCC Index. Below is a list of statements that other people with your illness have said are important. Circle one (1) number per line to indicate how true each statement has been for you during the past 7 days.

	**Not at all**	**A little bit**	**Some-what**	**Quite a bit**	**Very much**
I have pain	0	1	2	3	4
Pain interfered with my daily activities	0	1	2	3	4
I have pain in my back	0	1	2	3	4
I have discomfort or pain in my stomach area	0	1	2	3	4
I have a good appetite	0	1	2	3	4
I have control of my bowels	0	1	2	3	4
I urinate more frequently than usual	0	1	2	3	4
I have had chills	0	1	2	3	4
I have had fevers	0	1	2	3	4
I have had sweats	0	1	2	3	4
I feel lightheaded	0	1	2	3	4
I am bothered by blood in my urine	0	1	2	3	4
I am losing weight	0	1	2	3	4
I have trouble moving my bowels	0	1	2	3	4
I have been short of breath	0	1	2	3	4
I have felt weak	0	1	2	3	4
I have lacked appetite	0	1	2	3	4
I have difficulty urinating	0	1	2	3	4
I have had trouble sleeping	0	1	2	3	4
I feel fatigued	0	1	2	3	4
I have a lack of energy	0	1	2	3	4
I feel tired	0	1	2	3	4
I have trouble starting things because I am tired	0	1	2	3	4
I have trouble finishing things because I am tired	0	1	2	3	4
I have difficulty remembering things	0	1	2	3	4
I have trouble concentrating	0	1	2	3	4
I worry that my condition will get worse	0	1	2	3	4
I have emotional ups and downs	0	1	2	3	4
I feel depressed	0	1	2	3	4
I am able to enjoy life	0	1	2	3	4

## Discussion

The RCC Symptom Index was developed through a structured, iterative process that drew upon several information sources to identify the appropriate signs and symptoms. A broad range of physiological and psychological disease manifestations were identified and found relevant by a national, US cohort of RCC patients who were undoubtedly treated at different cancer centers.

Consistent with the medical literature review, the most common symptoms that initially led patients to seek medical attention were pain, fatigue, and urinary problems such as hematuria. Once diagnosed with RCC, both groups of patients – those with metastasized disease and those with localized disease who had undergone surgery for their renal cancer – indicated a rather high prevalence of symptoms. The symptoms most apparent among the localized RCC patients included irritability, pain, fatigue, worry, and sleep disturbance, while the metastatic-stage patients also included symptoms related to treatment and disease progression, including weakness and shortness of breath.

In a large-scale study of 335 patients with RCC randomized to subcutaneous interferon or medroxyprogesterone [8], the most frequently reported symptoms at week 4 assessed by the Rotterdam Symptom Checklist included tiredness (57%), lack of energy (53%), lack of appetite (33%), dry mouth (27%), and flu-like symptoms (nausea 15% and shivering 13%). Similar symptoms were reported in a study among 70 patients, 57 of whom had RCC, receiving doses of pegylated interferon alpha-2B administered subcutaneously for 4 weeks [13], including fatigue (38%), pain (34%), weight loss (28%), and dyspnea (21%). Smaller studies (i.e., those with 55 or fewer patients) produced similar results with respect to reported symptoms [11,12,14-17,19].

There are several limitations to this study. First, the RCC patients were a US convenience sample who self-reported their disease stage (localized vs. metastatic). It is likely that patients were unaware of their exact disease stage, and some patients who reported localized disease may have developed metastases following their nephrectomy [43]. The fact that several patients who reported localized RCC also indicated continued pain is suggestive of disease progression. In addition, while metastatic patients were asked about their symptoms in the last 7 days when completing the questionnaire, patients with localized disease were asked about symptoms prior to treatment since they can be asymptomatic after surgery [43]. Localized patients may not have recalled the symptom experience as accurately as possible if a shorter recall period had been possible. Similarly, symptom recall could become confused with post-surgical recovery, especially regarding pain and fatigue. However, anchoring of the symptom questions to what was undoubtedly a significant life event, i.e., renal cancer surgery, should have assisted with patient recall [52,53].

Our finding that metastatic patients did not rate pain as highly as localized patients was surprising given the literature associating pain with tumor progression [54]. Fewer than 25% of patients with metastatic disease reported "somewhat," "quite a bit," or "very much" for any single pain item. However, the lower frequency of reported pain among this subset of RCC patients may be due to concurrent pain treatment. Patients were not asked about their pain medications when being interviewed, and pain may have been better controlled among metastatic-stage patients. In the RCC population as a whole, pain was one of the most relevant symptoms; caregivers considered pain the most significant symptom. Finally, RCC patients who participated in the study were generally Caucasian and well-educated. These findings may have limited applicability to minority US populations and to those of other countries, although several of the studies appearing in the literature review contained these populations.

Despite these weaknesses, we believe that we were able to capture the relevant symptoms associated with kidney cancer since our findings are based on a synthesis of information from multiple sources: literature review, completed pilot questionnaires, structured patient and caregiver interviews, and clinician evaluation. The RCC Symptom Index is a brief, easily-understood, self-administered instrument that covers pain, shortness of breath, weakness, sleep, weight loss, cognitive functioning, fatigue, appetite, incontinence, other constitutional symptoms, and mood. The 7-day recall period is sufficiently short to maximize accurate patient recall while avoiding the possible loading effect of a "bad symptom day" or short-term flare caused by variation in symptom frequency, intensity, or duration. 

Systemic mechanisms such as cytokine dysregulation may underline cancer pathogenesis and explain the diversity of RCC symptoms [55,56].  Consequently, the content breadth of the Index may make it more sensitive to the full impact of systemic  therapies on RCC patient symptoms.  While this project was underway, a kidney cancer symptom scale was published: the Functional Assessment of Cancer Therapy-Kidney   Symptom Index (FKSI) [41]. Although the FKSI (15-items) is shorter than the RCC Symptom Index it has considerable overlap in content [41]. 

## Conclusion

This study systematically developed the content of a RCC patient symptom questionnaire using literature review, caregiver observation and above all, the perspective of patients with the disease. The questionnaire is brief yet comprehensive since it incorporates patient-identified signs and symptoms across the disease spectrum. Due to this diverse range of signs and symptoms, the Index may be a more sensitive measure of the disease than a general cancer symptom questionnaire. The Index can be used alone to measure RCC symptom burden with minimal respondent burden, or with other more general cancer instruments for a more comprehensive assessment of patient-reported outcomes.

The FKSI and RCC Symptom Index are currently undergoing comparative content validation in a cohort of 50 kidney cancer patients from several US clinical sites. This study has access to clinical information contained in the patient medical chart and will produce a refined version of a symptom questionnaire that should be applicable to RCC and kidney cancer patients. Construct validation research is needed on the performance of the resulting questionnaire, especially regarding its responsiveness. The applicability of the final version to multi-national populations requires further investigation. Last but not least, defining a minimal, clinically meaningful improvement, as well as worsening, during construct validation is critical to providing practicing physicians, medical directors and health policy makers with a useful HRQL tool.

## Competing interests

Don Robinson, Jr. and Jason Clark were employees of Centocor R&D, Inc. at the time of this study. All other coauthors received research support from Centocor R&D, Inc. for this study.

## Authors' contributions

All coauthors contributed equally to this manuscript. All authors read and approved the final manuscript.

## References

[B1] Bassil B, Dosoretz DE, Prout GR (1985). Validation of the tumor, nodes and metastasis classification of renal cell carcinoma. J Urol.

[B2] Golimbu M, Joshi P, Sperber A, Tessler A, Al-Askari S, Morales P (1986). Renal cell carcinoma: survival and prognostic factors. Urology.

[B3] Parker SL, Tong T, Bolden S, Wingo PA (1996). Cancer statistics, 1996. CA Cancer J Clin.

[B4] Elson PJ, Witte RS, Trump DL (1988). Prognostic factors for survival in patients with recurrent or metastatic renal cell carcinoma. Cancer Res.

[B5] Schmidinger M, Steger G, Wenzel C, Locker GJ, Budinsky AC, Brodowicz T, Kramer G, Marberger M, Zielinski CC (2001). Sequential administration of interferon-gamma, GM-CSF, and interleukin-2 in patients with metastatic renal cell carcinoma: results of a phase II trial. J Immunother.

[B6] Coppin C, Porzsolt F, Kumpf J, Coldman A, Wilt T (2000). Immunotherapy for advanced renal cell cancer. Cochrane Database Syst Rev.

[B7] Yang JC, Sherry RM, Steinberg SM, Topalian SL, Schwartzentruber DJ, Hwu P, Seipp CA, Rogers-Freezer L, Morton KE, White DE, Liewehr DJ, Merino MJ, Rosenberg SA (2003). Randomized study of high-dose and low-dose interleukin-2 in patients with metastatic renal cancer. J Clin Oncol.

[B8] (1999). Interferon-alpha and survival in metastatic renal carcinoma: early results of a randomised controlled trial. Medical Research Council Renal Cancer Collaborators. Lancet.

[B9] Motzer RJ, Murphy BA, Bacik J, Schwartz LH, Nanus DM, Mariani T, Loehrer P, Wilding G, Fairclough DL, Cella D, Mazumdar M (2000). Phase III trial of interferon alfa-2a with or without 13-cis-retinoic acid for patients with advanced renal cell carcinoma. J Clin Oncol.

[B10] Capuron L, Ravaud A, Gualde N, Bosmans E, Dantzer R, Maes M, Neveu PJ (2001). Association between immune activation and early depressive symptoms in cancer patients treated with interleukin-2-based therapy. Psychoneuroendocrinology.

[B11] Heinzer H, Mir TS, Huland E, Huland H (1999). Subjective and objective prospective, long-term analysis of quality of life during inhaled interleukin-2 immunotherapy. J Clin Oncol.

[B12] Atzpodien J, Kuchler T, Wandert T, Reitz M (2003). Rapid deterioration in quality of life during interleukin-2- and alpha-interferon-based home therapy of renal cell carcinoma is associated with a good outcome. Br J Cancer.

[B13] Bukowski R, Ernstoff MS, Gore ME, Nemunaitis JJ, Amato R, Gupta SK, Tendler CL (2002). Pegylated interferon alfa-2b treatment for patients with solid tumors: a phase I/II study. J Clin Oncol.

[B14] Cohen L, de Moor C, Parker PA, Amato RJ (2002). Quality of life in patients with metastatic renal cell carcinoma participating in a phase I trial of an autologous tumor-derived vaccine. Urol Oncol.

[B15] Joffe JK, Banks RE, Forbes MA, Hallam S, Jenkins A, Patel PM, Hall GD, Velikova G, Adams J, Crossley A, Johnson PW, Whicher JT, Selby PJ (1996). A phase II study of interferon-alpha, interleukin-2 and 5-fluorouracil in advanced renal carcinoma: clinical data and laboratory evidence of protease activation. Br J Urol.

[B16] Naglieri E, Lopez M, Lelli G, Morelli F, Amodio A, Di Tonno P, Gebbia N, Di Seri M, Chetri MC, Rizzo P, Abbate I, Casamassima A, Selvaggi FP, Colucci G (2002). Interleukin-2, interferon-alpha and medroxyprogesterone acetate in metastatic renal cell carcinoma. Anticancer Res.

[B17] Shamash J, Steele JP, Wilson P, Nystrom M, Ansell W, Oliver RT (2003). IPM chemotherapy in cytokine refractory renal cell cancer. Br J Cancer.

[B18] Stark D, Kiely M, Smith A, Velikova G, House A, Selby P (2002). Anxiety disorders in cancer patients: their nature, associations, and relation to quality of life. J Clin Oncol.

[B19] Whitehead RP, Lew D, Flanigan RC, Weiss GR, Roy V, Glode ML, Dakhil SR, Crawford ED (2002). Phase II trial of recombinant human interleukin-4 in patients with advanced renal cell carcinoma: a southwest oncology group study. J Immunother.

[B20] Williams G, Pazdur R, Temple R (2004). Assessing tumor-related signs and symptoms to support cancer drug approval. J Biopharm Stat.

[B21] Victorson D, Soni M, Cella D (2006). Meta-analysis of the correlation between radiographic tumor response and patient-reported outcomes. Cancer.

[B22] Kim HL, Belldegrun AS, Freitas DG, Bui MH, Han KR, Dorey FJ, Figlin RA (2003). Paraneoplastic signs and symptoms of renal cell carcinoma: implications for prognosis. J Urol.

[B23] Zisman A, Pantuck AJ, Dorey F, Chao DH, Gitlitz BJ, Moldawer N, Lazarovici D, deKernion JB, Figlin RA, Belldegrun AS (2002). Mathematical model to predict individual survival for patients with renal cell carcinoma. J Clin Oncol.

[B24] Schips L, Lipsky K, Zigeuner R, Salfellner M, Winkler S, Langner C, Rehak P, Pummer K, Hubmer G (2003). Impact of tumor-associated symptoms on the prognosis of patients with renal cell carcinoma: a single-center experience of 683 patients. Urology.

[B25] Patard JJ, Leray E, Cindolo L, Ficarra V, Rodriguez A, De La Taille A, Tostain J, Artibani W, Abbou CC, Guille F, Chopin DK, Lobel B (2004). Multi-institutional validation of a symptom based classification for renal cell carcinoma. J Urol.

[B26] Bacik J, Mazumdar M, Murphy BA, Fairclough DL, Eremenco S, Mariani T, Motzer RJ, Cella D (2004). The functional assessment of cancer therapy-BRM (FACT-BRM): a new tool for the assessment of quality of life in patients treated with biologic response modifiers. Qual Life Res.

[B27] de Haes JC, van Knippenberg FC, Neijt JP (1990). Measuring psychological and physical distress in cancer patients: structure and application of the Rotterdam Symptom Checklist. Br J Cancer.

[B28] Aaronson NK, Ahmedzai S, Bergman B, Bullinger M, Cull A, Duez NJ, Filiberti A, Flechtner H, Fleishman SB, de Haes JC, Kaasa S, Klee M, Osoba D, Razavi D, Rofe PB, Schraub S, Sneeuw K, Sullivan M, Takeda F, for the European Organization for Research and Treatment of Cancer Study Group on Quality of Life (1993). The European Organization for Research and Treatment of Cancer QLQ-C30: a quality-of-life instrument for use in international clinical trials in oncology. J Natl Cancer Inst.

[B29] (2000). Stedman's Medical Dictionary.

[B30] Pazdur R, Coia LR, Hoskins WJ, Wagman LD, eds (2002). Cancer Management: A multidisciplinary approach.

[B31] Wilson IB, Cleary PD (1995). Linking clinical variables with health-related quality of life. A conceptual model of patient outcomes. JAMA.

[B32] McHorney CA, Tarlov AR (1995). Individual-patient monitoring in clinical practice: are available health status surveys adequate?. Qual Life Res.

[B33] Sprangers MA, Moinpour CM, Moynihan TJ, Patrick DL, Revicki DA (2002). Assessing meaningful change in quality of life over time: a users' guide for clinicians. Mayo Clin Proc.

[B34] Webster K, Cella D, Yost K (2003). The Functional Assessment of Chronic Illness Therapy (FACIT) Measurement System: properties, applications, and interpretation. Health Qual Life Outcomes.

[B35] Chang CH, Cella D, Clarke S, Heinemann AW, Von Roenn JH, Harvey R (2003). Should symptoms be scaled for intensity, frequency, or both?. Palliat Support Care.

[B36] Hwang SS, Chang VT, Cogswell J, Kasimis BS (2002). Clinical relevance of fatigue levels in cancer patients at a Veterans Administration Medical Center. Cancer.

[B37] Mendoza TR, Wang XS, Cleeland CS, Morrissey M, Johnson BA, Wendt JK, Huber SL (1999). The rapid assessment of fatigue severity in cancer patients: use of the Brief Fatigue Inventory. Cancer.

[B38] Mallinson T, Cella D, Cashy J, Holzner B (2006). Giving meaning to measure: linking self-reported fatigue and function to performance of everyday activities. J Pain Symptom Manage.

[B39] Brown DJ, McMillan DC, Milroy R (2005). The correlation between fatigue, physical function, the systemic inflammatory response, and psychological distress in patients with advanced lung cancer. Cancer.

[B40] Jensen MP (2003). The validity and reliability of pain measures in adults with cancer. J Pain.

[B41] Cella D, Yount S, Du H, Dhanda R, Gondek K, Langefeld K, George J, Bro WP, Kelly C, Bukowski R (2006). Development and validation of the Functional Assessment of Cancer Therapy-Kidney Symptom Index (FKSI). J Support Oncol.

[B42] Russell CK, Gregory DM (2003). Evaluation of qualitative research studies. Evid Based Nurs.

[B43] Abeloff MD, Armitage JO, Niederhuber JE, Kastan MB, McKenna WG, eds (2004). Clinical Onoclogy.

[B44] Clark PE, Schover LR, Uzzo RG, Hafez KS, Rybicki LA, Novick AC (2001). Quality of life and psychological adaptation after surgical treatment for localized renal cell carcinoma: impact of the amount of remaining renal tissue. Urology.

[B45] Poulakis V, Witzsch U, de Vries R, Moeckel M, Becht E (2003). Quality of life after surgery for localized renal cell carcinoma: comparison between radical nephrectomy and nephron-sparing surgery. Urology.

[B46] Cella DF, Tulsky DS, Gray G, Sarafian B, Lloyd S, Linn E, Bonomi A, Silberman M, Yellen SB, Winicour P, Brannon J, Eckberg K, Purl S, Blendowski C, Goodman M, Barnicle M, Stewart I, McHale M, Bonomi P, Kaplan E, Taylor S, Thomas C, Harris J (1993). The Functional Assessment of Cancer Therapy scale: development and validation of the general measure. J Clin Oncol.

[B47] Esper P, Mo F, Chodak G, Sinner M, Cella D, Pienta KJ (1997). Measuring quality of life in men with prostate cancer using the functional assessment of cancer therapy-prostate instrument. Urology.

[B48] Cella DF, Bonomi AE, Lloyd SR, Tulsky DS, Kaplan E, Bonomi P (1995). Reliability and validity of the Functional Assessment of Cancer Therapy-Lung (FACT-L) quality of life instrument. Lung Cancer.

[B49] Cella D (1997). The Functional Assessment of Cancer Therapy-Anemia (FACT-An) Scale: a new tool for the assessment of outcomes in cancer anemia and fatigue. Semin Hematol.

[B50] Yellen SB, Cella DF, Webster K, Blendowski C, Kaplan E (1997). Measuring fatigue and other anemia-related symptoms with the Functional Assessment of Cancer Therapy (FACT) measurement system. J Pain Symptom Manage.

[B51] Heffernan N, Cella D, Webster K, Odom L, Martone M, Passik S, Bookbinder M, Fong Y, Jarnagin W, Blumgart L (2002). Measuring health-related quality of life in patients with hepatobiliary cancers: the functional assessment of cancer therapy-hepatobiliary questionnaire. J Clin Oncol.

[B52] Schaeffer NC, Presser S (2003). The science of asking questions. Ann Rev Sociology.

[B53] Groves RM, Fowler FJ, Couper MP, Lepkowski JM, Singer E, Tourangeau R (2004). Survey Methodology.

[B54] McGuire DB (2004). Occurrence of cancer pain. J Natl Cancer Inst Monogr.

[B55] Lee BN, Dantzer R, Langley KE, Bennett GJ, Dougherty PM, Dunn AJ, Meyers CA, Miller AH, Payne R, Reuben JM, Wang XS, Cleeland CS (2004). A cytokine-based neuroimmunologic mechanism of cancer-related symptoms. Neuroimmunomodulation.

[B56] Dodd MJ, Miaskowski C, Lee KA (2004). Occurrence of symptom clusters. J Natl Cancer Inst Monogr.

